# Supplementary material for: From theoretical models to practical deployment: A perspective and case study of opportunities and challenges in AI-driven cardiac auscultation research for low-income settings
Source: PLOS Digit Health. 2024 Dec 4;3(12):e0000437. doi: 10.1371/journal.pdig.0000437 (PMC11616830; doi:10.1371/journal.pdig.0000437)
Supplement: S1 Table — (PDF) [file pdig.0000437.s001.pdf]

S1 Table Indicative NASSS evaluation for the deployment of heart sound recordings in low-resource settings.

Domain	Question		Rating	
Condition or illness	What is the nature of the condition or illness?	1A	1	Well-characterized, well-understood, predictable
Condition or illness	What are the relevant sociocultural factors and comorbidities?	1B	2	Must be factored into care plan and service model
Technology	What are the key features of the technology?	2A	2	Not yet developed or fully interoperable; not 100% dependable
Technology	What kind of knowledge does the technology bring into play?	2B	1	Directly and transparently measures [changes in] the condition
Technology	What knowledge and/or support is required to use the technology?	2C	1	None or a simple set of instructions
Technology	What is the technology supply model?	2D	1	Generic, "plug and play" solutions requiring minimal customization; easily substitutable if supplier withdraws
Value proposition	What is the developer's business case for the technology (supply-side value)?	3A	2	Business case underdeveloped; potential risk to investors
Value proposition	What is its desirability, efficacy, safety, and cost effectiveness (demand-side value)?	3B	1	Technology is desirable for patients, effective, safe, and cost effective
Adopter system	What changes in staff roles, practices, and identities are implied?	4A	2	Existing staff must learn new skills and/or new staff be appointed
Adopter system	What is expected of the patient and is this achievable by, and acceptable to them?	4B	1	Nothing
Adopter system	What is assumed about the extended network of lay caregivers?	4C	2	Assumes a caregiver will be available when needed
Organization	What is the organization's capacity to innovate?	5A	na	na
Organization	How ready is the organization for this technology-supported change?	5B	na	na
Organization	How easy will the adoption and funding decision be?	5C	na	na
Organization	What changes will be needed in team interactions and routines?	5D	na	na
Organization	What work is involved in implementation and who will do it?	5E	na	na
Wider context	What is the political, economic, regulatory, professional (e.g., medicolegal), and sociocultural context for program rollout?	6A	na	na
Embedding and adaptation over time	How much scope is there for adapting and coevolving the technology and the service over time?	7A	na	na
Embedding and adaptation over time	How resilient is the organization to handling critical events and adapting to unforeseen eventualities?	7B	na	na
