# Supplementary material for: From theoretical models to practical deployment: A perspective and case study of opportunities and challenges in AI-driven cardiac auscultation research for low-income settings
Source: PLOS Digit Health. 2024 Dec 4;3(12):e0000437. doi: 10.1371/journal.pdig.0000437 (PMC11616830; doi:10.1371/journal.pdig.0000437)
Supplement: Attachment — Submitted filename: PDIG-D-23-00490-ReviewComments.pdf [file pdig.0000437.s002.pdf]

PDIG-D-23-00490

The paper, entitled 'From Theoretical Models to Practical Deployment: A Perspective and Case Study of Opportunities and Challenges in AI-driven Healthcare Research for Low-Income Settings' by Felix Heinrich Krones and Benjamin Walker, develops an AI model based on the Binary Bayesian ResNet. This model leverages overlapping log-mel spectrograms of patient heart sound recordings and integrates demographic data. Also, the authors assess the capacity of AI to address healthcare disparities. While the topic of this study is interesting, the paper lacks clarity due to its combination of a review article and a contribution paper, and it does not follow a well-defined narrative path to present its contribution. Unfortunately, I cannot recommend publishing the submitted manuscript in PLOS Digital Health before addressing the following points.

1. In section 1.2, as one of the main contributions of the submitted manuscript authors mentioned "we aim to broaden the discourse by focusing on more fundamental 63 error scores that are straightforward to interpret." but it is not clear how the presented results can support this aim.
2. In section 2.2, authors about how variation of facility and equipment and data collection can cause different performance of a model in different models. I suggest authors include how different training and basically human accuracy can play a role here. We have inter-rater and intra-rater variability so we basically have an upper bound for how accurate our "*ground truth"* labels are.
3. The study should include more comprehensive analysis on detecting murmur. In the current format its pretty much the same as "Dual Bayesian ResNet: A Deep Learning Approach to Heart Murmur Detection" published earlier as a proceeding of CinC 2022. I would like to see how the algorithm performs on classifying unclear cases.
4. As one of the main contributions of this paper, the authors mentioned" A case study on developing and evaluating a predictive AI model **tailored for low-income environments**," but it's not clear how the developed algorithm contributes to this setting.
5. Authors Allocated a good portion of the manuscript to AI deployment in healthcare (and not related to cardiovascular disease necessarily). I would recommend focusing exclusively on prior research or studies pertaining to cardiovascular disease. Additionally, it should be clarified how the results presented contribute to this research, as it is currently unclear.

Minor comments:

- Figure 1, does not have y axis and it is vague.
- AUC mentioned first in line 138 but the abbreviation was defined in line 341.
- Figure 3 caption, suggest avoiding "=" to show association specially when you used ":" in Figure 1.
- Figure 4, legend is not clear. I suggest using larger circles and color blind friendly colors.
- Figure 4, add labels for curves/lines in the plot.
- Section names are not well defined.

- Deployment challenges (NASSS). *why NASS in parentheses?*
- 3.4.2 (Bayesian) Neural Network. *why parentheses?*
- 4.4 Model robustness . *Assessing model robustness*
- *…*
