# Supplementary material for: From theoretical models to practical deployment: A perspective and case study of opportunities and challenges in AI-driven cardiac auscultation research for low-income settings
Source: PLOS Digit Health. 2024 Dec 4;3(12):e0000437. doi: 10.1371/journal.pdig.0000437 (PMC11616830; doi:10.1371/journal.pdig.0000437)
Supplement: Attachment — Submitted filename: Answer_to_reviewers.docx [file pdig.0000437.s003.docx]

Felix Krones

Oxford Internet Institute

University of Oxford

[felix.krones@oii.ox.ac.uk](mailto:felix.krones@oii.ox.ac.uk)

12 August 2024

Matthew A Reyna, Ph.D.

Guest Editor

PLOS Digital Health

Dear Dr Reyna,

We would like to thank you and the reviewers for the comments, which we think have now improved our paper. We addressed the comments from the reviewers and summarised our changes.

Please find below a point-by-point response to each of the reviewers’ comments. Furthermore, our comments have been highlighted in the revised manuscript to track all the changes that we have made.

Sincerely,

Felix Krones (on behalf of all authors)

# Reviewer 1

Reviewer’s comment 1: “*While the scope of the analysis of the application of AI-based method for healthcare in low-income countries seems quite broad, the experimental evidence provided in the paper is focused on the specific case of cardiac auscultation. In this sense, I would suggest to narrow the scope of the discussion to this specific case, thus altering slightly the title of the work, and, most importantly, providing a more detailed discussion on the opportunities and limitation associated specifically to cardiac auscultation for cardiovascular disease screening.*”

Our response/changes: Thank you a lot for this comment! We agree, a more narrow discussion while going more into detail on cardiac auscultation makes the paper much clearer and more focused. We changed the title of the work, shortened and re-wrote the entire discussion section of the paper, with a clear focus on cardiac auscultation

- New title: From Theoretical Models to Practical Deployment: A Perspective and Case Study of Opportunities and Challenges in AI-driven Cardiac Auscultation Research for Low-Income Settings
- Discussion: We removed two sections completely (“Ripple-effect risks” and “Practical deployment challenges in low-income settings”). We added a section “Cardiac Auscultation” and re-wrote the other sections to make the focus much clearer.

Reviewer’s comment 2: “*When evaluating the generalization capabilities of the different deep learning methods considered, it would be interesting to provide a more systematic set of results, that could involve other public datasets on cardiac auscultation. As a first step, it would be interesting, for example, to repeat the proposed analysis when training on the PhysioNet 2016 dataset and testing on the PhysioNet 2022 dataset, thus providing a more varied training population that do not encompass mostly pediatric subjects.*”

Our response/changes: Thank you again, this is a nice idea! We followed the suggestion and trained and tested our model in both ways, on the 2016 and 2022 data. Table 8 summarises the results and the corresponding section 4.4 discusses the results in more detail. Moreover, we also added the comparison to the Yaseen dataset.

Reviewer’s comment 3: “*More details regarding what "effective implementation and conscientious design" (as mentioned in page 2) could be provided in the discussion.*”

Our response/changes: As we rewrote the entire discussion section to address comment 1 we hope that this comment got also solved. If this comment still applies also to the new version, we are happy to make additional changes.

Reviewer’s comment 4: “*In page 3, the authors mentioned that a binary classification task is considered, motivated by the fact that the proposed method is used as a prescreening tool. However, a significant portion of the heart sound recordings labeled as "unclear" in the PhysioNet 2022 dataset is affected by a severe amount of noise. In this sense, it would be advisable to provide a discussion on the impact of signal quality assessment (as mentioned later in the work) and the possible consequences of treating as abnormal also noisy recordings.*”

Our response/changes: Thank you, we tried to make this limitation clearer in the beginning of the work and also in the discussion. Moreover, we tried to change the narrative of the work to better highlight that our focus was on the outcome classification (for which often only two labels were available) and not so much on the murmur task for which the unclear labels were available.

Reviewer’s comment 5: “*Multimodal data is shown to guarantee improved accuracy and generalizability. However, it would be interesting to showcase more in details what is the impact of the demographic data used for classification, in terms of what kind of information is used to steer the classifier output and which cases are more benefitted by the presence of multimodal data.*”

Our response/changes: This is a very interesting idea to investigate which kind of multimodal data are helpful in which situation. While we thought this was out of scope for this paper, we actually started to work on a separate paper that looks specifically on that: When are multimodal data most helpful? We would be very happy to also hand in this work at this journal to get a review on it.

Reviewer’s comment 6: “*In Section 4.5, it is stated that ensuring standardized data collection is critical. In this sense, it would be interesting to reflect on the actual feasibility of this step, especially when considering low-income settings, and to reflect on the interplay between this aspect and unsupervised domain alignment techniques mentioned later in the paper.*”

Our response/changes: Thank you! We tried to address the first point as part of the literature review in the new section 2.4 and again together with the second point on the alignment in the new discussion section 5.3.

Reviewer’s comment 7: “*It would be interesting to include a discussion about the role of interpretable and explainable models in bridging the gap towards practical applications, overviewing the current state-of-the-art of such solutions for the problem of cardiac auscultation.*”

Our response/changes: We added a paragraph on that to section 5.3 “Limitations and Future Research”.

Reviewer’s comment 1 Minor: “*I would suggest to avoid the term POC "monitoring" and use instead POC "screening", as the task considered in the case of cardiac auscultation is that of disease detection and not follow-up.*”

Our response/changes: Thank you, good point, done!

Reviewer’s comment 2 Minor: “*In page 15, the authors claim that the congruence between the challenge scores and performance obtained in the reserved portion of the training data suggests that their approach effectively generalizes across diverse datasets. I think that this statement should be toned down, as the challenge test data was also collected from the same screening campaigns, in the same conditions as the training set. Therefore, performance on the challenge test set is not representative of performance on diverse datasets.*”

Our response/changes: This makes sense and we changed the wording of the entire section 4.3.

# Reviewer 2

Reviewer’s comment 1: “*In section 1.2, as one of the main contributions of the submitted manuscript authors mentioned “we aim to broaden the discourse by focusing on more fundamental error scores that are straightforward to interpret.” but it is not clear how the presented results can support this aim.*”

Our response/changes: Thank you! We agree, especially our abstract and introduction were not very clear on that. We rewrote the entire abstract and section 1.2 and hope this makes the work clearer now.

Reviewer’s comment 2: “*In section 2.2, authors about how variation of facility and equipment and data collection can cause different performance of a model in different models. I suggest authors include how different training and basically human accuracy can play a role here. We have inter-rater and intra-rater variability so we basically have an upper bound for how accurate our “ground truth” labels are.*”

Our response/changes: Again, thank you! We rewrote the entire section 2.2 and (together with the comments of reviewer 1) the entire discussion section, also discussing this limitation more focused. We hope this makes it clearer.

Reviewer’s comment 3: “*The study should include more comprehensive analysis on detecting murmur. In the current format its pretty much the same as “Dual Bayesian ResNet: A Deep Learning Approach to Heart Murmur Detection” published earlier as a proceeding of CinC 2022. I would like to see how the algorithm performs on classifying unclear cases.*”

Our response/changes: By changing the narrative of the entire paper we tried to make the difference to the previous paper much clearer, especially that in here we wanted to highlight challenges towards generalisability. The point on investigating unclear cases is a very interesting point and together with comment 5 of reviewer 1 we are currently working on a new paper that investigates the effect of different data for the different cases in detail and hope to publish it also in this journal very soon.

Reviewer’s comment 4: “*As one of the main contributions of this paper, the authors mentioned” A case study on developing and evaluating a predictive AI model tailored for low-income environments,” but it's not clear how the developed algorithm contributes to this setting.*”

Our response/changes: We agree that this point was not very clear from our initial work and aligns with some comments from reviewer 1. While rewriting especially the abstract, the introduction, section 2 and the discussion, we hope to have made this point much clearer now.

Reviewer’s comment 5: “*Authors Allocated a good portion of the manuscript to AI deployment in healthcare (and not related to cardiovascular disease necessarily). I would recommend focusing exclusively on prior research or studies pertaining to cardiovascular disease. Additionally, it should be clarified how the results presented contribute to this research, as it is currently unclear.*”

Our response/changes: Thank you, this very much aligns and extends on reviewer 1’s first comment. As mentioned above, we rewrote the entire discussion section to make this clearer. In addition, we basically changed the whole section 2 to now have a much clearer separation between section 2 and the discussion, to make clearer what is more general deployment focus and what is specifically on cardiovascular diseases.

Reviewer’s comment 1 Minor: “*Figure 1, does not have y axis and it is vague.*”

Our response/changes: We agree, the figure did not add much and we removed it.

Reviewer’s comment 2 Minor: “*AUC mentioned first in line 138 but the abbreviation was defined in line 341.*”

Our response/changes: Good point, we fixed it.

Reviewer’s comment 3 Minor: “*Figure 3 caption, suggest avoiding “=” to show association specially when you used “:” in Figure 1.*”

Our response/changes: This is aligned now, thank you!

Reviewer’s comment 4 Minor: “*Figure 4, legend is not clear. I suggest using larger circles and color blind friendly colors.*”

Our response/changes: Thank you, very helpful! We increased the circle size, added labels to the lines and switched to the colorblind seaborn palette.

Reviewer’s comment 5 Minor: “*Figure 4, add labels for curves/lines in the plot.*”

Our response/changes: Thank you, very helpful! Together with the comment above we increased the circle size, added labels to the lines and switched to the colorblind seaborn palette.

Reviewer’s comment 6 Minor: “*Section names are not well defined.*”

Our response/changes: Thank you! We changed almost all section titles to make it clearer what the corresponding sections are about.

Reviewer’s comment 7 Minor: “*Deployment challenges (NASSS). why NASS in parentheses?*”

Our response/changes: Good point, we changed it, thank you!

Reviewer’s comment 8 Minor: “*3.4.2 (Bayesian) Neural Network. why parentheses?*”

Our response/changes: Again, changed, similar to the point above. Thank you!

Reviewer’s comment 9 Minor: “*Model robustness . Assessing model robustness -...*”

Our response/changes: We were unsure what this comment meant but maybe it got resolved by addressing all the others. If not, happy to address it.

# Reviewer 3

Reviewer’s comment 1: “*The survey alternates between a broad survey of using AI to improve healthcare in LMICs and the more targeted application of using AI to screen of PCGs for heart murmurs. Due to the broad scope and difficulty of a comprehensive survey, I recommend conducting a narrower survey and noting when the observations extend more broadly instead of conducting a broad survey and providing narrower examples. In general, the attempt to tie together a broad survey with a specific application is admirable but imperfectly executed.*”

Our response/changes: Thank you a lot for this comment! We agree and think this goes very much along with the first comment of the first reviewer. We especially rewrote the entire abstract, introduction, section 2 and the discussion to narrow it down and to make the story line clearer.

Reviewer’s comment 2: “*The use of other datasets, i.e., the PhysioNet Challenge 2016 training set, as an out-of-sample holdout set to assess generalizability is helpful. I would recommend switching these datasets as well, i.e., use the PhysioNet Challenge 2016 training set as a training set and the PhysioNet Challenge 2022 training set as a holdout set, to more comprehensively assess the generalizability of this approach to an underrepresented population.*”

Our response/changes: Thank you! Again, I think this aligns very much with the second comment of the first reviewer and we implemented the suggestion by training and testing in both ways. Table 8 summarises the results and the corresponding section 4.4 discusses the results in more detail. Moreover, we also added the comparison to the Yaseen dataset.

Reviewer’s comment 3: “*Several words, such as "Binary", "Artificial Intelligence", and "Area Under the Curve", are capitalized but probably should not be under the journal's style guide.*”

Our response/changes: Thank you, we tried to be consistent now with it.

Reviewer’s comment 4: “*Some references need additional information to identify the cited resources and for compliance with the journal's style guide.*”

Our response/changes: Thank you! We went over each reference and adjusted them to meet the journal guide and the vancouver citation style as mentioned in the guide.

# Additional changes

We used the opportunity and revised the whole paper, making the flow and focus clearer and improved the language.
